# Identification of three small nucleolar RNAs (snoRNAs) as potential prognostic markers in diffuse large B‐cell lymphoma

**DOI:** 10.1002/cam4.5115

**Published:** 2022-08-16

**Authors:** Mei‐wei Li, Feng‐xiang Huang, Zu‐cheng Xie, Hao‐yuan Hong, Qing‐yuan Xu, Zhi‐gang Peng

**Affiliations:** ^1^ Department of Medical Oncology First Affiliated Hospital of Guangxi Medical University Nanning Guangxi Zhuang Autonomous Region P. R. China

**Keywords:** diffuse large B‐cell lymphoma, microarray profiles, risk model, small nucleolar RNAs

## Abstract

**Background:**

Diffuse large B‐cell lymphoma (DLBCL) is a non‐Hodgkin lymphoma with high mortality rates. Small nucleolar RNAs (snoRNAs) are tumor‐specific biological markers, but there are few studies on the role of snoRNAs in DLBCL.

**Materials and Methods:**

Survival‐related snoRNAs were selected to construct a specific snoRNA‐based signature via computational analyses (Cox regression and independent prognostic analyses) to predict the prognosis of DLBCL patients. To assist in clinical applications, a nomogram was built by combining the risk model and other independent prognostic factors. Pathway analysis, gene ontology analysis, transcription factor enrichment, protein–protein interactions, and single nucleotide variant analysis were used to explore the potential biological mechanisms of co‐expressed genes.

**Results:**

Twelve prognosis‐correlated snoRNAs were selected from the DLBCL patient cohort of microarray profiles, and a three‐snoRNA signature consisting of SNORD1A, SNORA60, and SNORA66 was constructed. DLBCL patients could be divided into high‐risk and low‐risk cohorts using the risk model, and the high‐risk group and activated B cell‐like (ABC) type DLBCL were linked with disappointing survival. In addition, SNORD1A co‐expressed genes were inseparably linked to the biological functions of the ribosome and mitochondria. Potential transcriptional regulatory networks have also been identified. *MYC* and *RPL10A* were the most mutated SNORD1A co‐expressed genes in DLBCL.

**Conclusion:**

Put together, our findings explored the potential biological effects of snoRNAs in DLBCL, and provided a new predictor for DLBCL prediction.

## Graphical abstract

A comprehensive analysis was performed to select significant survival‐related snoRNAs in DLBCL. Collectively, a prognostic signature based on three snoRNAs (SNORD1A, SNORA60, and SNORA66) was established for DLBCL patients. Also, we found snoRNA co‐expressed genes that were mostly involved in ribosome‐correlated pathways and processes, and the interaction of ribosomal proteins (RPL23, RPL10A) and p53.

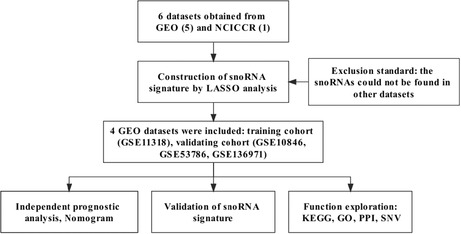

## 1 INTRODUCTION

Diffuse large B‐cell lymphoma (DLBCL) is an aggressively malignant hyperplastic disease, accounting for approximately one‐third of all non‐Hodgkin lymphoma (NHL) cases worldwide.^1^ According to gene expression profiling, DLBCL is classified into three types: germinal center B‐cell like (GCB), activated B‐cell like (ABC), and unclassifiable, among which ABC DLBCL has a lower 3‐year survival rate of approximately 40%–50%, compared to approximately 75% of GCB DLBCL.^2^, ^3^ Although the complete response rate for DLBCL patients is over 60%, after receiving the standard treatment protocols, R‐CHOP (cyclophosphamide, doxorubicin, vincristine, and prednisone with rituximab), approximately one‐third of patients suffer from relapse or refractory disease due to chemoresistance, with a median survival time of <6 months.^4^ The dominant clinical measurement, International Prognostic Index (IPI), can predict clinical outcomes, but does not distinguish biological heterogeneity.^1^ Therefore, investigating prognostic biological molecules, as well as alternative treatment strategies for DLBCL is of great significance.

With the current next‐generation sequencing and computational techniques evolving rapidly, the critical roles of non‐coding RNAs (ncRNAs), including microRNAs (miRNAs), long ncRNAs (lncRNAs), circular RNAs, piwi‐associated RNAs, and small nucleolar RNAs in the regulation of tumorigenesis and tumor progression have come to light.^5^, ^6^, ^7^ Among ncRNAs, miRNAs and lncRNAs are the most extensively studied as vital factors in the diagnosis, treatment, and prognosis of DLBCL^8^, ^9^, ^10^; however, less attention has been paid to other classes of ncRNAs, particularly snoRNAs, which are comparable to miRNAs in terms of diversity and number.

snoRNAs are mainly found in the eukaryotic cell nuclei, 60–300 base pairs long, and contain conserved structural elements.^11^ Based on their structural components and biological roles, snoRNAs are classified into H/ACA snoRNAs (SNORA) and C/D box snoRNAs (SNORD). SNORAs guide the dioxymethylation of nucleotides, whereas SNORDs are responsible for pseudouridylation.^11^, ^12^ Additionally, current data suggest that snoRNAs are involved in modifying snRNAs, tRNAs, and even mRNAs, and associated with the pathogenesis of multiple malignancies, suggesting their potential as prognostic biomarkers.^13^, ^14^, ^15^ Because of their carcinogenic roles, several snoRNA host genes, *SNHG12*,^16^ *SNHG14*,^17^, ^18^ and *SNHG16* ^19^ have been recognized as promising therapeutic targets in DLBCL. Among these, high expression of *SNHG12* was found to correlate with poor prognosis in DLBCL patients.^16^ Despite these findings, the clinical value of snoRNAs in DLBCL remains to be determined.

This study aimed to screen snoRNA predictors based on microarray profile data and construct an independent and specific snoRNA‐based signature using a series of computational analyses. To explore the underlying mechanism of snoRNAs in DLBCL, the genes co‐expressed with snoRNAs in DLBCL were identified through analyses of pathways, gene ontology, transcriptional regulation and mutations.

## 2 MATERIALS AND METHODS

### 2.1 Data acquisition and processing

The human snoRNA list was extracted from the snoDB (http://scottgroup.med.usherbrooke.ca/snoDB/) database^20^ and provided details of human snoRNAs integrating current literature and similar database resources, such as snoRNABase, snOPY, and snoRNA Atlas. Using “lymphoma” as the search term in Gene Expression Omnibus (GEO) database, prognostic snoRNA profiles of DLBCL were gathered. RNA sequencing (RNA‐seq) data for DLBCL were downloaded from The Cancer Genome Atlas (TCGA). Expression data of snoRNAs in DLBCL, patient survival information, and other clinical parameters were downloaded for analysis, and all expression data were log2 normalized. Datasets without sufficient survival information, as well as datasets with prognostically relevant patients and fewer than 50 cases were excluded. The expression levels of snoRNAs were examined in two or more samples, and the average expression value was used for subsequent analyses. snoRNA expression and relevant clinical features were matched to the human snoRNA list obtained from snoDB.

### 2.2 Construction and validation of snoRNA prediction models

All the following calculations were performed using R 4.0.2. In the construction of the microarray profile, univariate Cox regression analysis was utilized to screen out snoRNAs related to overall survival (OS) in DLBCL patients. Significant survival‐related snoRNAs (*p* < 0.05) were included for the next analyses. Then the LASSO regression analysis is widely applied to variable selection and shrinkage for minimization of overfitting risk and can be used for prognostic model establishment.^21^ Thus, we constructed an optimal prognostic snoRNA signature in DLBCL via 1000 cross‐validation iterations in LASSO regression and obtained regression coefficients (*β*). To calculate the risk scores to confirm microarray profiles, the following formula was used: the risk score was equal to the sum of (snoRNA_1_ expression × *β*_snoRNA1_ + snoRNA_2_ expression × *β*_snoRNA2_ + ...... + snoRNA_*n*_ expression × *β*_snoRNAn_). The in‐between hazard score was regarded as the cut‐off point. The Survival package in R 4.0.2 was used for subsequent survival analyses. To estimate the difference in OS between the high‐ and low‐ risk groups, the Kaplan–Meier (K‐M) method was used. Using SurvivalROC R package to plot receiver operating characteristic curve, also known as ROC curve, predictive efficiency of the snoRNA model was determined. The area under the curve (AUC) was computed as well. To ensure the robustness and reliability of the risk model, an exclusion standard was made for the obtained models: the snoRNAs of the model could not be found in other datasets.

### 2.3 Exploration of the clinical value of risk model

Clinical correlation analyses of the risk model and corresponding clinicopathological features were conducted to explore the clinical value of the prognostic signature. The chi‐square test was applied for clinical correlation analysis and the relevant cluster heat map was generated. The possibilities of risk score for predictive model and clinical indicators as significant prognostic markers were evaluated using independent prognostic analyses, via “forestplot” R package.

### 2.4 Establishment of prognostic nomogram and calibration

The risk model and independent clinicopathological parameters were combined to construct a nomogram, an individualized scoring system for prognosis that is conducive to prognosis prediction in DLBCL patients. Higher total scores were suggestive of worse clinical outcomes. The construction of nomogram was generated through “rms” package in R. Also, to test reliability, calibration analysis of the nomogram was conducted.

### 2.5 Functional annotation of genes co‐expressed with candidate snoRNAs

SnoRNAs, which are mainly embedded in introns of protein‐ or non protein‐coding genes, have been implicated in the process of posttranscriptional regulation of mRNA, snRNA, and tRNA.^22^ The co‐expression relationships between candidate snoRNAs and encoding genes were explored using Pearson's correlation analysis. Genes with an absolute value of correlation coefficient > 0.4 (moderate correlation) were selected for further functional examination. These mRNAs were subsequently subjected to functional enrichment analysis, containing Kyoto Encyclopedia of Genes and Genomes (KEGG) and Gene Ontology (GO) analyses, via DAVID 6.8 (Database for Annotation, Visualization, and Integrated Discovery version 6.8, https://david.ncifcrf.gov/home.jsp). The statistically significant annotation KEGG and GO terms (*p* < 0.05) were visualized using the ggplot2 package in R. To assess the association among these genes, protein–protein interaction networks (PPI) were drawn using high‐confidence interactions (interaction score ≥ 0.7) in the STRING database (https://string‐db.org/). The genes ranked as top 10 according to degree values were selected as hub genes using Cytoscape software.

### 2.6 Transcriptional regulation of snoRNA‐related genes in DLBCL

Transcription factors (TFs) that were potentially involved in regulating snoRNA‐related genes and their biological processes were also predicted in DAVID v6.8. In addition, TF‐hub gene networks were predicted and visualized using Cytoscape software. Chromatin immunoprecipitation (ChIP)‐seq was adopted to explore the underlying transcriptional regulation of TFs and snoRNA‐related genes, which downloaded from Cistrome Data Browser (http://cistrome.org/db/#/).

### 2.7 Single nucleotide variant analysis of snoRNA‐related genes in DLBCL

Maf data of single nucleotide variant (SNV) for DLBCL in the TCGA database was acquired from the UCSC online tool (https://xena.ucsc.edu/), which was calculated using the varscan method. R package “maftools” was used to analyze the mutations of SNORD1A co‐expression genes. A waterfall plot was used to visualize the SNV distribution.

## 3 RESULTS

### 3.1 Prognostic risk model based on snoRNAs for DLBCL

**FIGURE 1:**
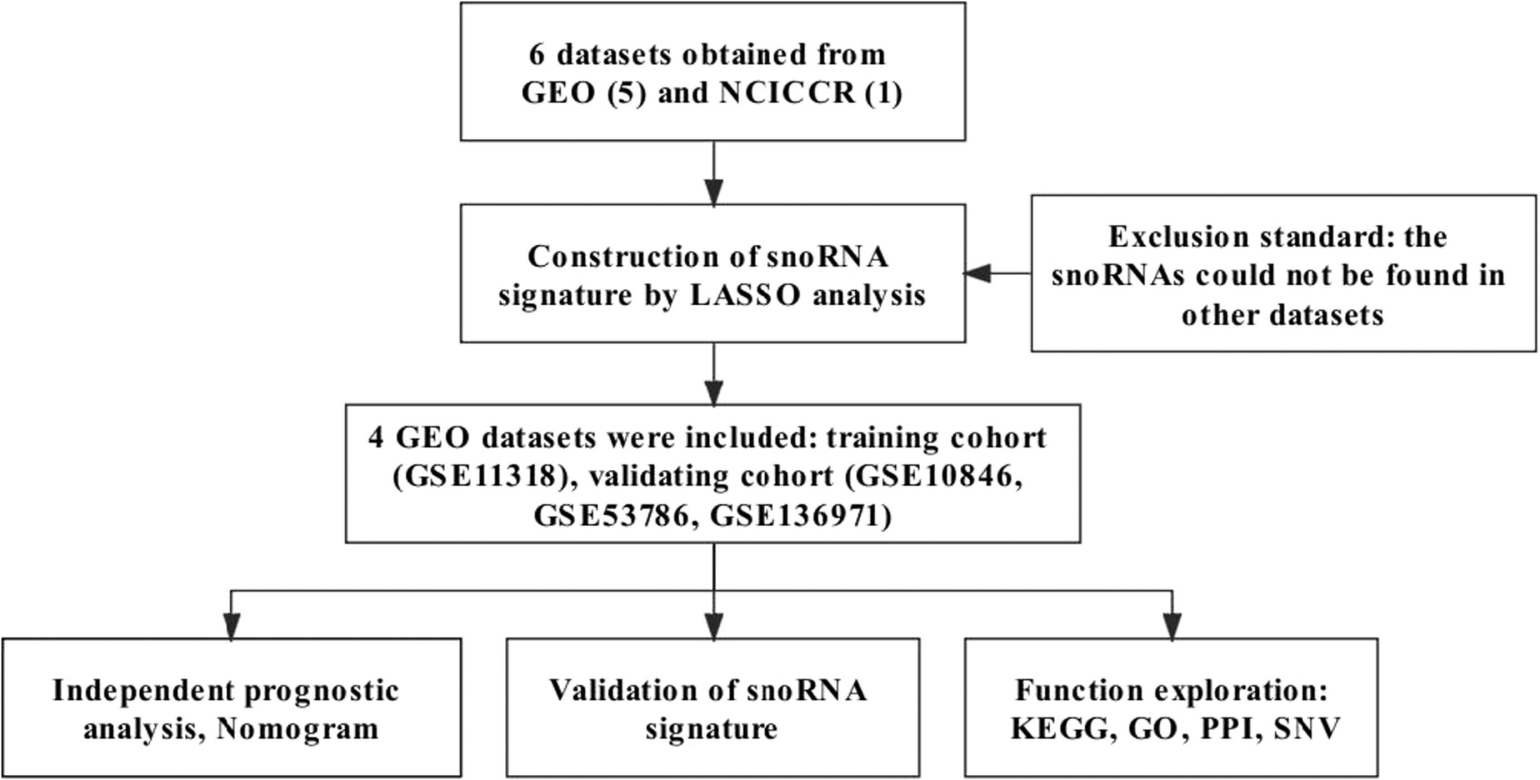
Flow diagram of the study design

Figure 1 illustrated the study design. A total of 751 human snoRNAs were acquired from the SnoDB online database (Tables S1 and S2), from which duplicated snoRNAs were deleted. In total, we obtained six datasets for subsequent analyses, including five microarray profiles (GSE11318, GSE10846, GSE53786, GSE31312, GSE136971) from the GEO database and one RNAseq dataset (NCICCR: the National Cancer Institute Center for Cancer Research) from the TCGA database, as shown in Table S2.

We then constructed prognostic snoRNA signatures based on the above datasets using Cox regression analyses. After matching the list of human snoRNAs from SnoDB to expression matrixes of the six datasets, we finally obtained 74 snoRNAs, 75 snoRNAs, 74 snoRNAs, 22 snoRNAs, 75 snoRNAs and 112 snoRNAs for prognosis analysis in GSE11318, GSE10846, GSE53786, GSE31312, GSE136971 and NCICCR, respectively. Randomly, when one dataset worked as the training cohort, the other datasets worked as the validated cohorts. Using univariate and LASSO Cox regression analyses, risk models for six training cohorts were preliminarily established, including GSE11318 (SNORD1A, SNORA60, and SNORA66), GSE10846 (SNORA66, SNORD35B, and SNORA70), GSE53786 (SNORD84, SNORD4A, and SNORD1A), GSE31312 (SNORA68, SNORD8, and SCARNA13), GSE136971 (SNORA67, SNORA11D, and SNORA37) and NCICCR (SNORD104, SNORD116‐18, and SNORD51). All *p* values of the six prognostic risk models in the K‐M analysis were lower than 0.05, indicating significant statistical significance (Figures S1 and S2). More details regarding the six prognostic signatures for DLBCL are provided in Table S2. After excluding the unqualified risk models, we finally included the risk models consisting of SNORD1A, SNORA60, and SNORA66 in GSE11318 for subsequent analyses.

### 3.2 Clinical parameters for the included DLBCL patients

Four microarray profiles (GSE11318, GSE10846, GSE53786, and GSE136971) were included in the prognosis risk model construction (184 cases in GSE11318), and validation (380 cases in GSE10846, 76 cases in GSE5378, and 214 cases in GSE136971). A total of 854 DLBCL patients underwent follow‐up survival analysis. Complete clinical information for 147, 320, 59, and 214 patients in GSE11318, GSE10846, GSE53786, and GSE136971, respectively (Table 1).

**TABLE 1:** Clinical features of patients with DLBCL in microarray profiles

Clinical features	GSE11318	GSE10846	GSE53786	GSE136971
Gender				
Male	67	141	22	113
Female	80	179	37	101
Age				
≤65	82	191	30	145
>65	65	129	29	69
Sate				
Dead	80	118	26	46
Alive	67	202	33	168
Subtype				
ABC DLBCL	62	128	27	79
GCB DLBCL	62	146	21	81
Unclassified DLBCL	23	46	11	54
Stage				
1	21	56	9	29
2	49	99	15	42
3	31	71	15	34
4	46	94	20	109
ECOG performance status				
0	34	76	13	NA
1	80	174	24	NA
2	25	48	16	NA
3	8	20	5	NA
4	0	2	1	NA
Number of extranodal sites				
0	122	198	31	NA
1	25	95	21	NA
2	0	17	5	NA
3	0	7	2	NA
4	0	2	0	NA
5	0	1	0	NA

### 3.3 Prognostic signature consisting of SNORD1A, SNORA60, and SNORA66 in DLBCL

**FIGURE 2:**
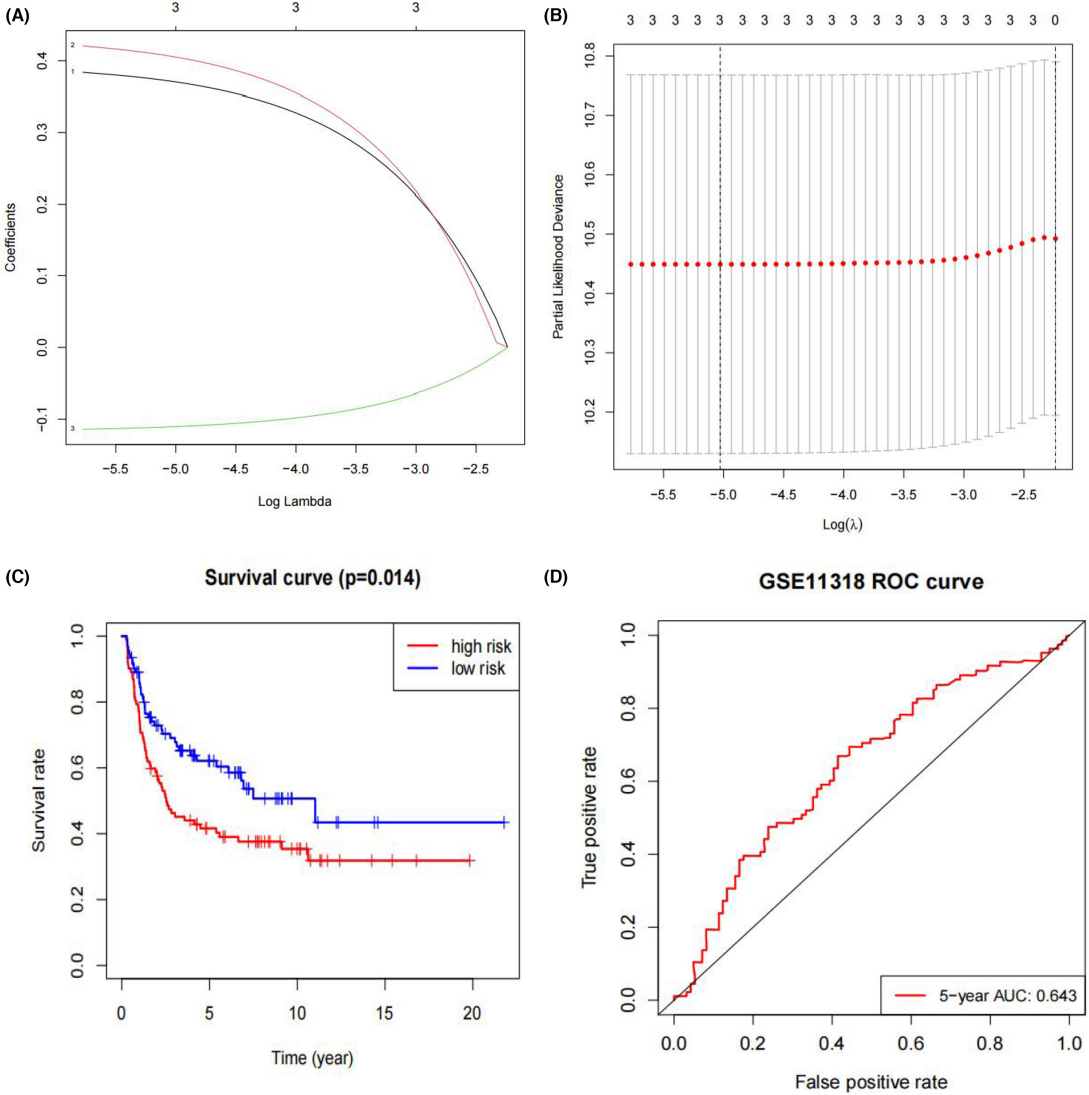
Construction of prognostic snoRNA signature for DLBCL. (A) LASSO coefficient profiles of three included risk snoRNAs. (B) Tuning parameter (lambda) selection cross‐validation in LASSO model. The optimal values were chosen by using the minimum and 1‐SE criteria, and then the dotted vertical lines were drawn. (C) K‐M survival analysis of the three‐snoRNA signature in GSE11318. (D) ROC analysis for three‐snoRNA prognostic signature.

In GSE11318, we obtained 74 snoRNAs for prognostic analysis after matching the human snoRNAs list from SnoDB to the expression matrix. The 74 snoRNAs were then subjected to univariate Cox regression analysis for assessment of prognostic value of snoRNAs in DLBCL. A total of 12 prognosis‐correlated snoRNAs were screened out (*p* < 0.05, Table 2). LASSO regression was then conducted to establish a predictive prognostic model for three candidate snoRNAs: SNORD1A (snoRNA, C/D box 1A; chr17: 76561633_76561706), SNORA60 (snoRNA, H/ACA box 60; chr20: 38449369_38449504), and SNORA66 (snoRNA, H/ACA box 66; chr1: 92840719_92840851) (Figure 2). The division of DLBCL patients was determined according to the medium risk score (cutoff point). In addition, high‐hazard patients had worse prognosis than low‐hazard patients (*p* = 0.014, Figure 2). The 1‐, 3‐, and 5‐year survival rates of high‐hazard patients were 0.750, 0.452, and 0.403, respectively, compared to 0.856, 0.678, and 0.604, respectively, for low‐hazard patients. Individuals with high‐risk scores had a propensity for disappointing outcomes. In addition, the AUC for this signature was 0.663 and 0.643 at 3 and 5 years, respectively, suggesting definite sensitivity and specificity for the prediction of overall survival (Figure 2).

**TABLE 2:** Summary of univariate analyses for 12 prognosis snoRNAs in DLBCL

ID	HR	95%CI LL	95%CI UL	*p* value
SNORA73A	1.6509	1.2605	2.1621	0.0003
SNORD19B	1.9635	1.3457	2.8650	0.0005
SNORD88C	1.5077	1.1536	1.9705	0.0026
SCARNA16	1.6921	1.1960	2.3939	0.0030
SNORA17	1.7154	1.1972	2.4578	0.0033
SNORD110	1.7700	1.1422	2.7429	0.0106
SNORA4	1.6357	1.1146	2.4004	0.0119
SNORA71A	1.3226	1.0372	1.6867	0.0242
SNORD44	1.4727	1.0269	2.1119	0.0353
SNORD1A	1.6027	1.0297	2.4946	0.0366
SNORA60	1.8385	1.0261	3.2939	0.0407
SNORA66	0.8685	0.7580	0.9951	0.0423

Abbreviations: CI, confidence interval; HR, hazard ratio; LL, lower limit; UL, upper limit.

**FIGURE 3:**
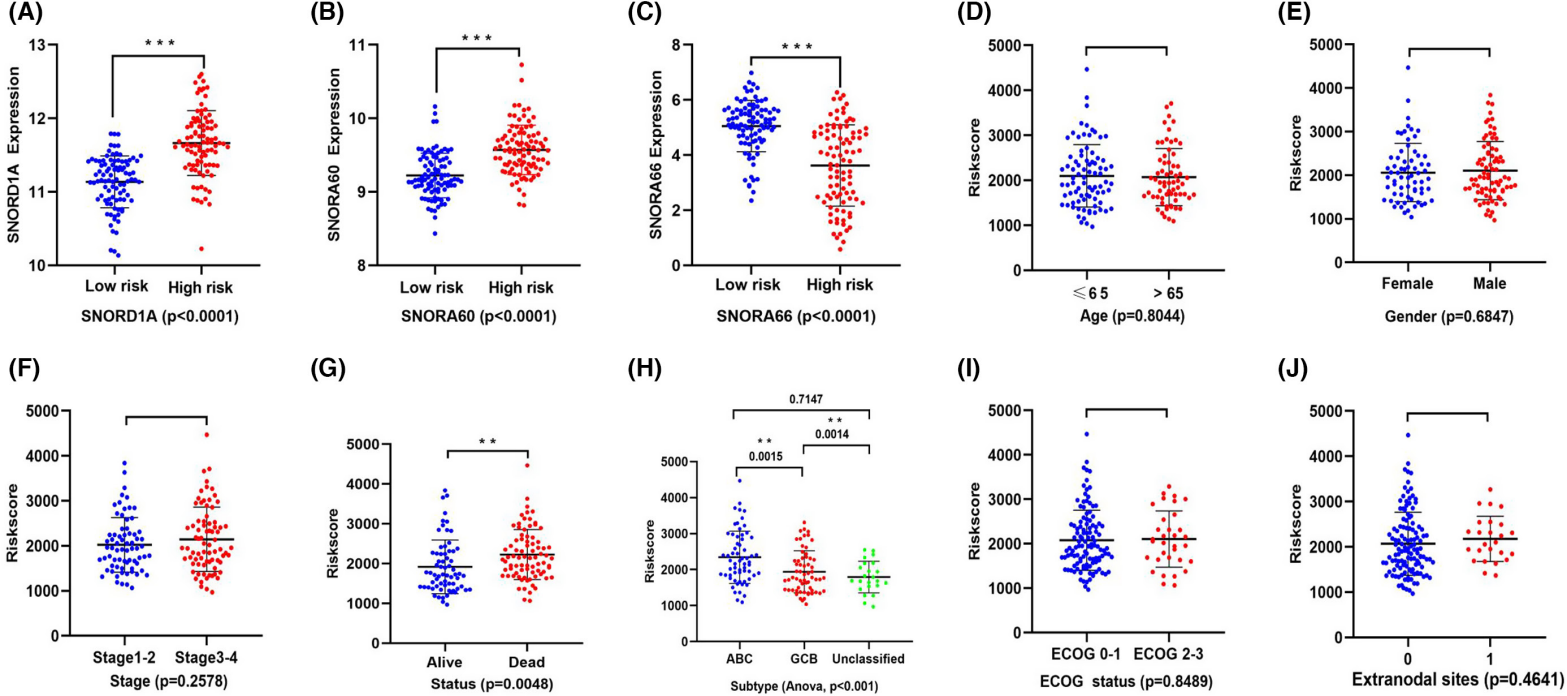
(A, B) SNORD1A and SNORA60 were both up‐regulated in high‐risk cohort. (C) SNORA66 was down‐regulated in high‐risk cohort. (D–J) Relationship of risk‐score and clinicopathological characteristics in DLBCL patients: (D) Age, (E) Gender, (F) Stage, (G) Status, (H) Subtype, (I) ECOG status, (J) Extranodal sites. Statistical significance was set at *p*‐value < 0.05. **p value < 0.05, ***p value < 0.001.

SNORD1A and SNORA60 showed higher expression levels in DLBCL tissues, than SNORA66. The average expression levels of SNORD1A, SNORA60, and SNORA66 were 11.4274, 9.4125, and 4.1978, respectively. SNORD1A and SNORA60 were up‐regulated in the high‐risk scores cohorts. In contrast, the up‐regulation of SNORA66 was observed in the low‐risk cohort (Figure 3). Univariate Cox regression analysis suggested that SNORD1A (HR = 1.6027, *p* = 0.0366) and SNORA60 (HR = 1.8385, *p* = 0.0407) were harmful factors for DLBCL patients, whereas SNORA66 (HR = 0.8685, *p* = 0.0423) was recognized as a protective factor (Table 2).

### 3.4 Correlation of prognostic risk model and clinicopathological characteristics in DLBCL

**FIGURE 4:**
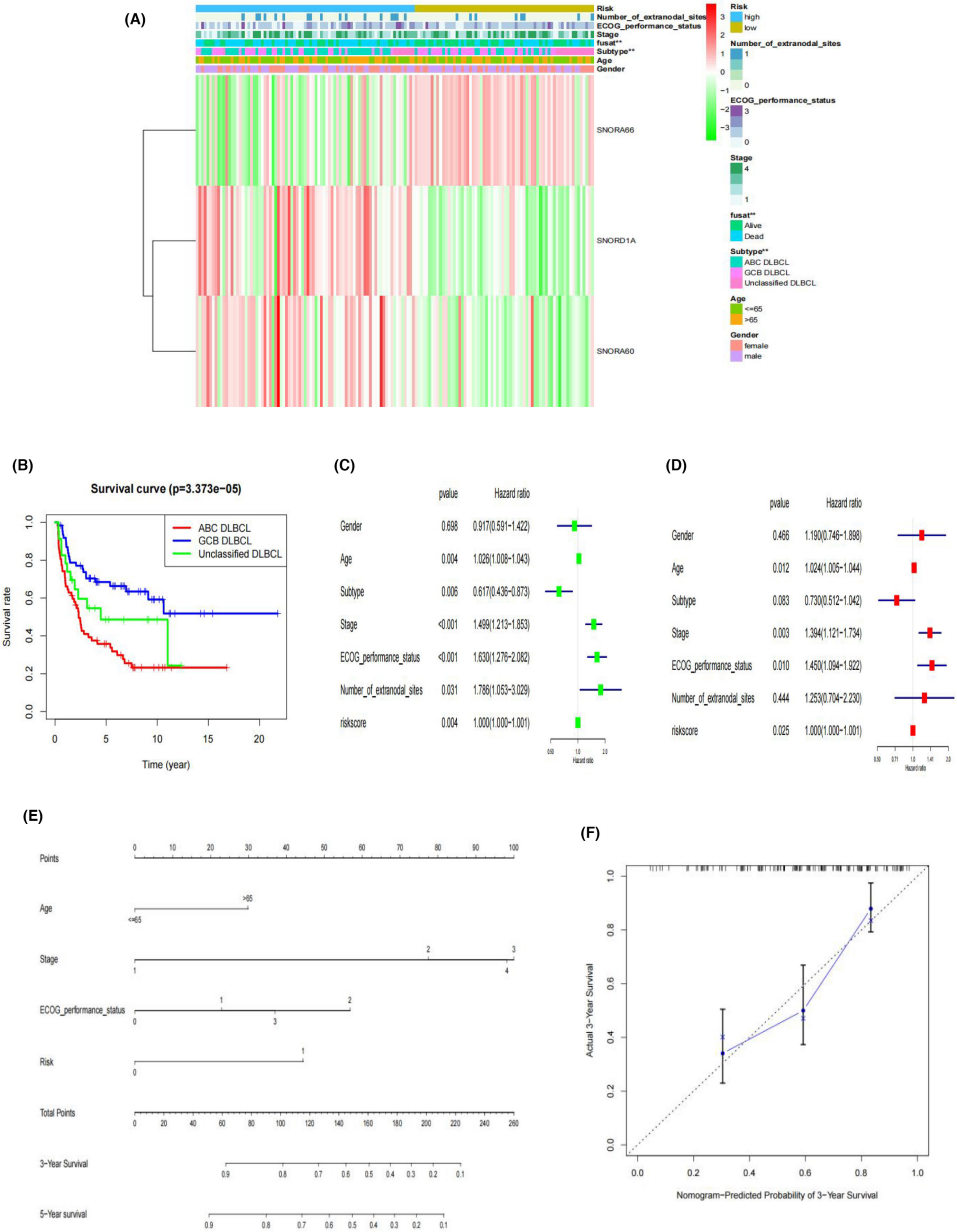
(A) Correlation of candidate snoRNAs expression, risk‐score, and the clinicopathologic information shown in the cluster heatmap. Red color for snoRNA refers to high risk score, whereas green color for snoRNA means low risk score. The levels of color referred to the value risk score. The other colors matched to different features that were noted in the figure legends. (B) K‐M survival analysis of different DLBCL subtypes in GSE11318. (C, D) Independent prognostic analysis of snoRNA risk model in DLBCL: Forest plot of univariate and multivariable cox analysis. (E) Nomogram for independent prognostic factors (three‐snoRNA signature, age, stage and ECOG performance status) to predict 3‐, or 5‐year survival probability in DLBCL. Each variable was assigned a score according to its contribution to the prognosis of DLBCL (Age: <=65, 0 points; >65, 30 points. Stage: 1, 0 points; 2, 77 points; 3, 100 points; 4, 98 points. ECOG performance status: 0, 0 points; 1, 23 points; 2, 57 points; 3, 37 points; Risk model: 0, 0 points; 1, 44points). Then, total points were obtained by adding single point of each variable and used for predicting the probability of the clinical ending for individual or clinical outcome. (F) Calibration curves of nomogram‐predicted probability for 3‐year overall survival

First, risk scores were compared across subgroups (Figure 3), suggesting that high risk patients were highly likely to have ABC‐type DLBCL. Moreover, we arranged DLBCL patients using their risk scores to further explore their association with clinicopathological characteristics. The elevated risk score was obviously significant linked with the DLBCL subtype (*p* < 0.001, Figure 3). However, the risk scores and other clinical parameters (gender, age, ECOG score, stage, and number of extranodal sites) were not significantly associated (*p* > 0.05, Figures 3 and 4). The prognostic value of the snoRNA model was explored for different DLBCL subtypes. We found that ABC‐type DLBCL patients had a worse survival than GCB‐type and unclassified DLBCL patients in the training cohort (GSE11318, *p* < 0.001), shown as in Figure 4.

### 3.5 Integration analysis of candidate prognostic risk model

Assessment of the possibility of the three‐snoRNA signature and other clinicopathological features as independent risk factors for DLBCL was conducted through independent prognostic analysis. According to univariate analysis, the three‐snoRNA model, age, subtype, stage, ECOG performance status, as well as number of extranodal sites had a close relationship with the state of DLBCL patients (*p* < 0.05, Figure 4). In multivariable Cox regression analysis, the risk signature, age, stage, and ECOG performance status were four independent predictors of prognosis in DLBCL (Figure 4). Overall, the three‐snoRNA risk model could be considered as an independent risk predictor to assess the prediction effect. The nomogram, a quantitative scoring method, was then plotted by combining the four independent risk factors (three‐snoRNA signature, age, stage, and ECOG performance status) to predict survival probability for DLBCL patients (Figure 4). Each variable was assigned a score according to its contribution to DLBCL prognosis (Age: ≤65, 0 points; >65, 30 points. Stage: 1, 0 points; 2, 77 points; 3, 100 points; 4, 98 points. ECOG performance status: 0, 0 points; 1, 23 points; 2, 57 points; 3, 37 points; Risk model: 0, 0 points; 1, 44points). Then, the total points were obtained by adding a single point of each variable and used to predict the probability of the clinical outcome for an individual or clinical outcome. Patients with higher total points tended to have shorter survival. According to the calibration curves, then prediction accuracy of this nomogram was close to that of the ideal model (Figure 4).

### 3.6 Validation of the prognostic snoRNA signature in other microarrary profiles

**FIGURE 5:**
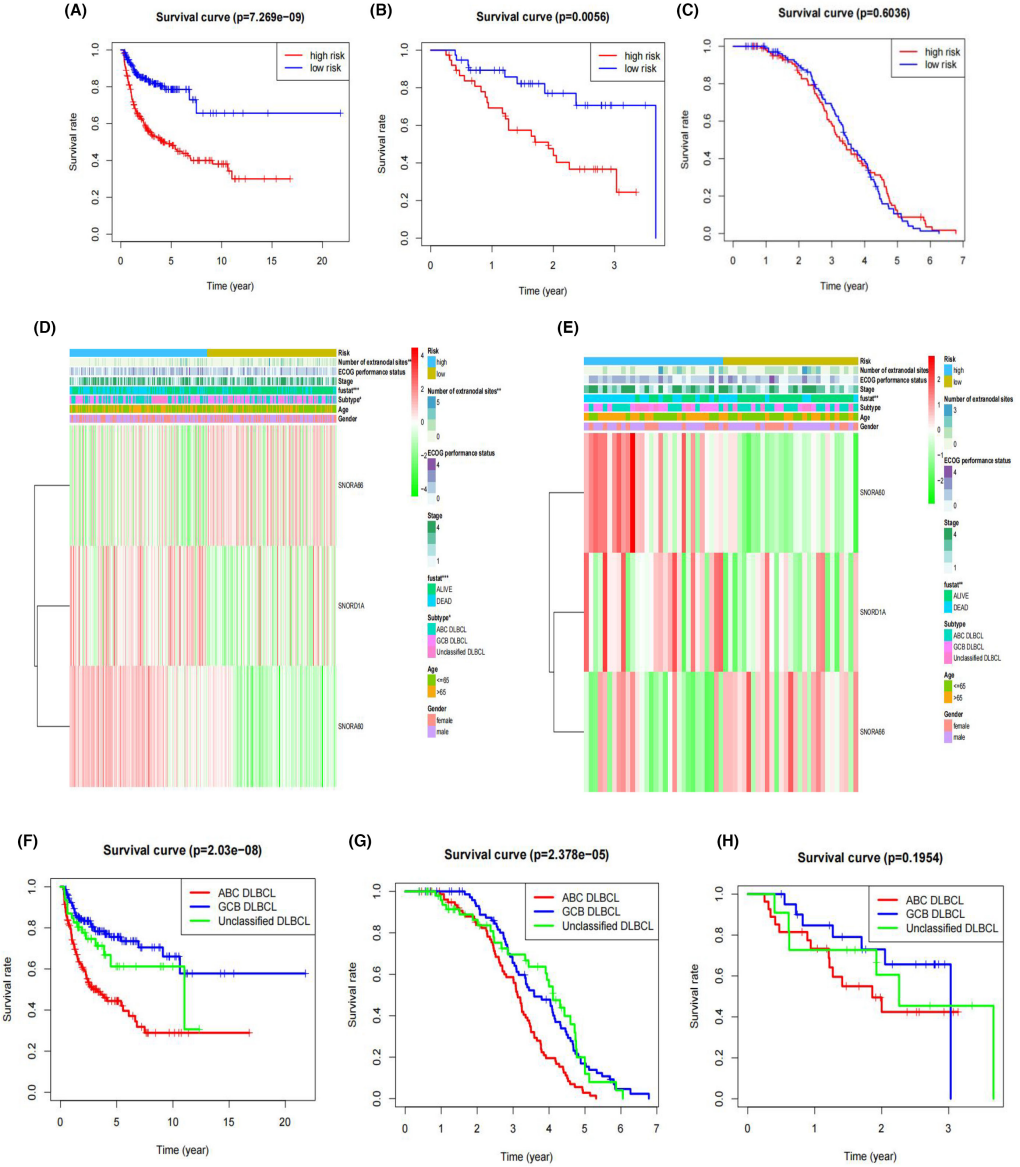
Validation of prognostic snoRNA signature for DLBCL. (A–C) K‐M survival curve of overall survival based on the three‐snoRNA signature in GSE10846, GSE53786, GSE136971. (D, E) The correlation of candidate snoRNAs and clinical features in GSE10846 and GSE53786. Red color for snoRNA refers to high risk score, while green color for snoRNA means low risk score. The levels of color referred to the value risk score. The other colors matched to different features that were noted in the figure legends. (F–H) K‐M survival analysis of different DLBCL subtypes in GSE10846, GSE136971, and GSE53786

Similar results for prognostic prediction, clinical correlation, and independent analysis were also observed in three validation series: GSE10846, GSE53786, and GSE136971. The formula was: (expression of SNORD1A) × (0.3709) + (expression of SNORA60) × (0.4059) + (expression of SNORA66) × (−0.1106). We validated our snoRNA signature in both GSE10846 (*p* < 0.0001) and GSE53786 (*p* = 0.0056) and found that a high‐risk score was correlated with worse prognosis in DLBCL patients (Figure 5). For example, in GSE10846, 1‐, 3‐, and 5‐ year survival rates were 0.776, 0.539, and 0.481 for predicting OS in the high‐risk cohort, and 0.918, 0.826, and 0.786 in the low‐risk cohort. Although the *p*‐value of GSE136971 (*p* = 0.6036) was greater than 0.05, we could also discover the trend of low‐risk patients with a better outcome.

Next, we selected GSE10846 and GSE53786 to verify the relationship between the risk model and clinical features. Similar to GSE11318, the risk model was correlated with the subtype and number of extranodal sites in GSE10846 but was not associated with any clinicopathological features in GSE53786 (Figure 5). Both series indicated an independent role of the three‐snoRNA signature in DLBCL (Figure S2). Worse prognosis was found to be linked to some clinical features, such as older age, GCB subtype, advanced stage, and high ECOG performance status in GSE10846. As for prognosis in different DLBCL subtypes, ABC type DLBCL patients showed worse survival in the validation cohorts (GSE10846 and GSE136971) compared to GCB type and unclassified DLBCL patients, and all the *p* values were <0.001 (Figure 5). A trend of having a lower overall survival rate was found in ABC type DLBCL patients for the GSE53786 dataset (*p* = 0.1954, Figure 5).

### 3.7 Functional examination of the predictive prognostic snoRNAs in DLBCL

snoRNAs and their correlated mRNAs are reported to be involved in post‐transcriptional processes that trigger tumor development. Correlation analysis was performed to determine the correlations of snoRNAs with their co‐expressed protein‐coding genes, and moderately co‐expressed mRNAs (|correlation coefficient| > 0.4) were selected for the next function exploration. We acquired 356 and 10 co‐expression genes for SNORD1A and SNORA60, respectively, but no qualified co‐expression genes were identified for SNORA66.

**FIGURE 6:**
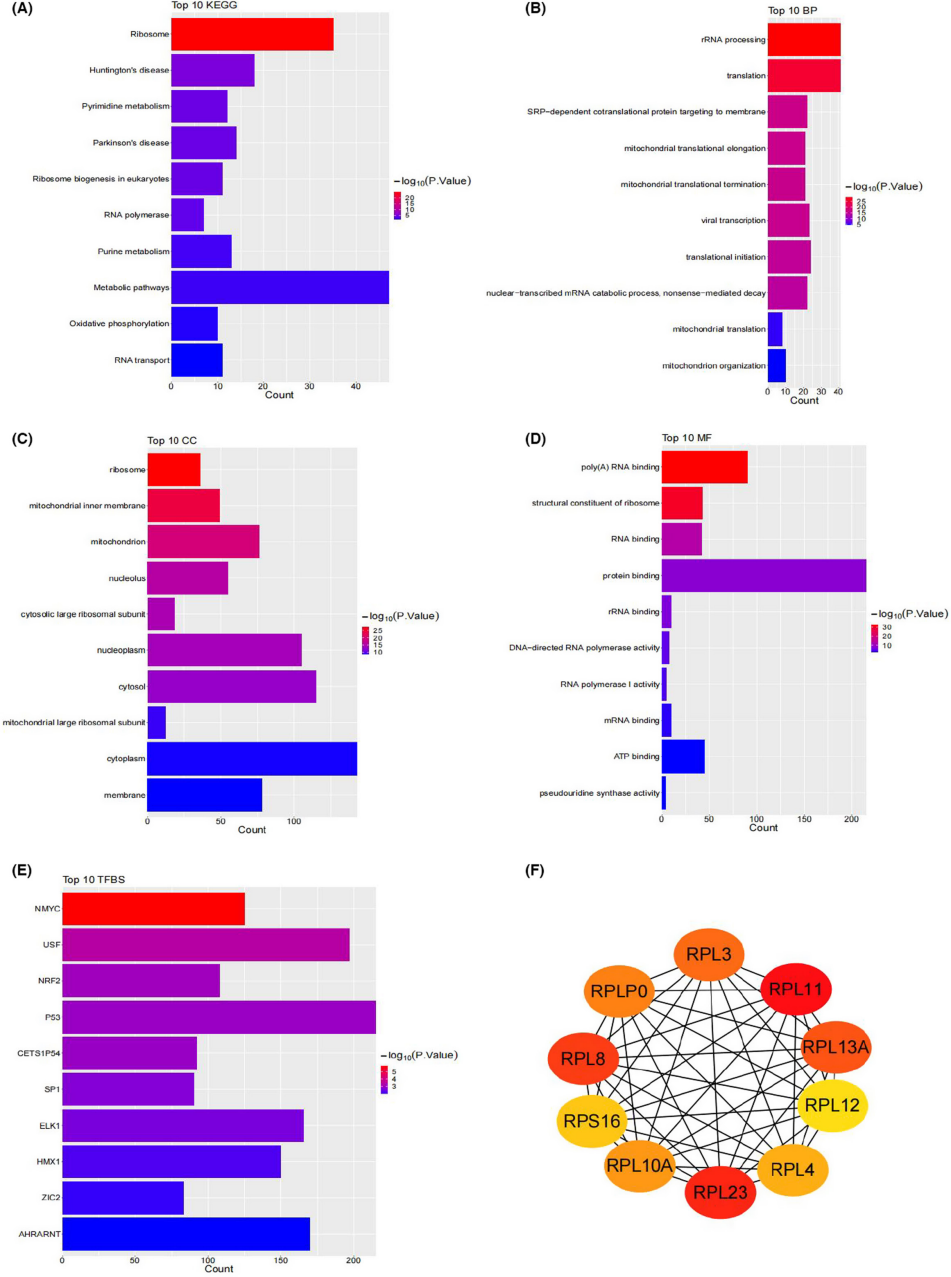
The potential mechanism of snoRNAs co‐expressed genes for SNORD1A. (A) The top 10 significant KEGG pathways. (B–D) The top 10 significant GO terms. (E) The top 10 significant transcription factors (TF). (F) The potential hub genes. To make the figures more visual, the *p* values of KEGG and GO terms were −log10 normalized using ggplot2 package in R.

KEGG and GO enrichment analyses were conducted in David, suggesting that SNORD1A co‐expression genes are mostly involved in Ribosome, Huntington's disease, Pyrimidine metabolism, Parkinson's disease, Ribosome biogenesis in eukaryotes, RNA polymerase, Purine metabolism, Metabolic pathways, Oxidative phosphorylation, RNA transport for KEGG; rRNA processing and some biological activities related to mitochondrion for BP; Ribosome and Mitochondrion for CC; Poly(A) RNA binding and Structural constituent of ribosome for MF; *NMYC*, *USF* and *NRF2* for TF (Figure 6). SNORD1A co‐expression genes were enriched in several snoRNA‐relevant terms, as shown in Table 3. What's more, according to the results of PPI analysis using the String and MCC methods of cytoHubba in cytoscape, *RPL11*, *RPL23*, *RPL8*, *RPL13A*, *RPL3*, *RPLP0*, *RPL10A*, *RPL4*, *RPS16*, and *RPL12* may be the top 10 hub genes for SNORD1A. The aforementioned networks are shown in Figure 6.

**TABLE 3:** SNORD1A co‐expressed mRNAs involved in biological activities of snoRNA

Term	Count	*p*‐Value	Genes
BP_SRP‐dependent cotranslational protein targeting to membrane	22	2.64E‐17	*RPL4*, *RPL3*, *RPL23*, *RPS5*, *RPL12*, *RPL11*, *RPLP0*, *RPL13A*, *RPSA*, *SRP68*, *RPL10A*, *RPL8*, *RPL7A*, *RPS16*, *RPL14*, *RPL24*, *SRPRB*, *RPL35*, *RPL13*, *RPS2*, *RPL29*, *UBA52*
BP_snRNA pseudouridine synthesis	3	0.0010	*DKC1*, *NHP2*, *NOP10*
CC_small nucleolar ribonucleoprotein complex	4	0.0014	*LSM7*, *NHP2*, *NOP10*, *SNRPB*
CC_box H/ACA scaRNP complex	3	0.0018	*DKC1*, *NHP2*, *NOP10*
CC_box H/ACA telomerase RNP complex	3	0.0018	*DKC1*, *NHP2*, *NOP10*
CC_box H/ACA snoRNP complex	3	0.0018	*DKC1*, *NHP2*, *NOP10*
MF_box H/ACA snoRNA binding	3	0.0020	*DKC1*, *NHP2*, *NOP10*
CC_U4/U6 x U5 tri‐snRNP complex	4	0.0074	*PRPF4*, *SNRPD2*, *PPIH*, *SNRPB*
BP_snRNA transcription from RNA polymerase II promoter	6	0.0095	*SNAPC4*, *POLR2E*, *POLR2G*, *POLR2H*, *POLR2I*, *POLR2L*
BP_small nuclear ribonucleoprotein complex	3	0.0353	*SNRPD2*, *SLU7*, *SNRPB*

Abbreviations: BP, biological process; CC, cellular component; MF, molecular function.

### 3.8 Construction and validation of a potential transcriptional regulatory network

**FIGURE 7:**
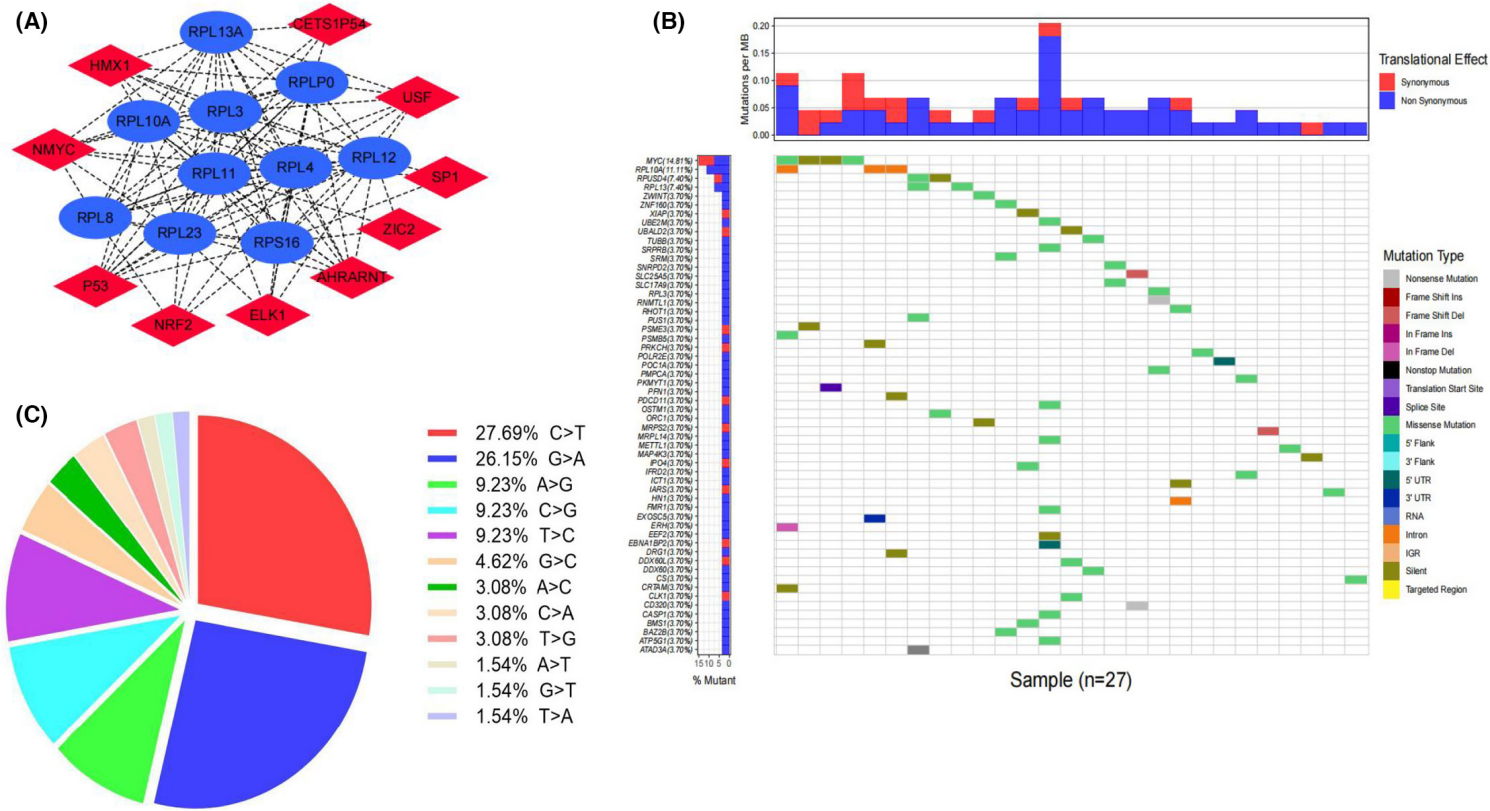
(A) The network of transcription factors and hub genes for SNORD1A.Red color refers to transcription factors, whereas blue color refers to hub genes. (B) Heat map of the mutations for SNORD1A co‐expressed genes in TCGA‐DLBCL cohort. Different colors matched to different mutation types that were noted in the figure legends. Each row represents the mutations of a SNORD1A co‐expressed gene in 27 DLBCL samples, whereas each column represents the mutations of SNORD1A co‐expressed genes in a DLBCL sample. The gene with the highest mutations frequency rate was *MYC* (up to 14.81%) and *RPL10A* (up to 11.11%). (C) The pie graph shows the classification of mutations. C>T mutations (27.69%) occurred in most of the samples, followed by G>A (26.15%).

We constructed a potential interactive network of transcription factors and hub genes according to the enrichment results of transcription factors and their relevant genes in David using cytoscape software (Figure 7). Additionally, we checked specific ChIP‐seq data for tissue/blood of lymphoma in humans or mus musculus using the Cistrome Data Browser to identify the binding sites of potential transcription factors and hub genes (Table 4). The binding sites with a combined score > 1.0 were recognized to be significant, such as *p53*‐*RPL23* (Mus musculus, Lymphoma, coordinate: chr11:97777525–97,782,438, score: 1.4505), *p53*‐*RPL10A* (Mus musculus, Lymphoma, coordinate: chr17:28328564–28,331,032, score: 1.1760), *p53*‐*RPS16* (Mus musculus, Lymphoma, coordinate: chr7:28350688–28,352,697, score: 1.3798), *p53*‐*RPL12* (Mus musculus, Lymphoma, coordinate: chr2:32961711–32,964,044, score: 1.3535), and *ZIC2*‐*RPL12* (Homo sapiens, Lymphoma, coordinate: chr9:127447673–127,451,405, score: 1.1500).

**TABLE 4:** Potential binding sites of transcription factors and hub genes in Lymphoma from CHIP‐seq data in Cistrome DB

snoRNA	TF	PMID	Species	Tissue	Disease	Target	Coordinate	Score
SNORD1A	*p53*	28,092,679	Mus musculus	Lymph node	Lymphoma	*RPL11*	chr4:136049946–136,053,355	0.6190
						*RPL11*	chr4:136049353–136,053,392	0.7085
						*RPL23*	chr11:97777525–97,782,438	1.4505
						*RPL13A*	chr7:45125562–45,128,744	0.7275
						*RPL3*	chr15:80077780–80,083,405	0.7823
						*RPLP0*	chr5:115559466–115,563,728	0.5230
						*RPL10A*	chr17:28328564–28,331,032	1.1760
						*RPL4*	chr9:64173386–64,178,561	0.8345
						*RPS16*	chr7:28350688–28,352,697	1.3798
						*RPL12*	chr2:32961711–32,964,044	1.3535
SNORD1A	*ZIC2*	28,835,494	Homo sapiens	Peritoneal effusion	Lymphoma	*RPL11*	chr1:23691805–23,696,834	0.0030
						*RPS16*	chr19:39433136–39,435,948	0.2090
						*RPL12*	chr9:127447673–127,451,405	1.1500

### 3.9 Mutation analysis of snoRNA‐related genes in DLBCL

A total of 37 DLBCL samples with mutations were identified in the TCGA‐DLBCL cohort. For SNORD1A co‐expression genes, approximately 72.97% of DLBCL patient samples (27/37) carried mutations, of which missense mutations were of the highest frequency (Figure 7). C > T mutations (27.69%) occurred in most samples, followed by G > A (26.15%), (Figure 7). The genes with the highest mutation frequency rates were *MYC* (up to 14.81%) and *RPL10A* (up to 11.11%), as shown in Figure 7.

## 4 DISCUSSION

Approximately 70% of DLBCL patients are diagnosed terminally, and 20 to 25% of patients receiving the standard care R‐CHOP still suffer from relapse, especially within the initial 2 years.^1^ Thus, identifying novel and effective predictors is crucial for high‐risk DLBCL patients. In clinical practice, constructing a risk assessment model based on prognostic indicators is crucial for diverse malignancies. Jiang et al. created a new immune checkpoint‐ and hypoxia‐based independent risk model for acute myeloid leukemia (AML), facilitating the development of new targets for AML therapies.^23^, ^24^ Hu et al. combined clinical variables and pharmacogenomic genes to construct a risk model to improve drug resistance in DLBCL patients.^25^ Risk score models based on prognostic genes related to metabolism,^26^ pyroptosis,^27^ and ferroptosis^28^ have been established for DLBCL, providing novel ideas for individualized therapeutic strategies in DLBCL patients. The past few years have witnessed the potential of several snoRNAs as prognostic molecular biomarkers in various cancers, and predictive risk signatures based on certain snoRNAs have been found in carcinomas of the lung,^29^, ^30^ liver,^31^ stomach,^32^ colon,^33^ kidney,^34^, ^35^ bladder,^36^ head and neck,^37^ and eyes,^38^ as well as sarcoma,^39^ and T‐cell lymphoma.^40^ The current literature has reported the potential oncogenic functions of several snoRNAs in the carcinogenesis of DLBCL,^16^, ^17^, ^18^, ^19^ and SNHG12 might be a risk factor for the clinical outcome of DLBCL patients. However, to date, no report exists on a similar prognostic risk model for snoRNAs in DLBCL.

In our study, survival‐associated snoRNAs were screened in a DLBCL patient cohort of microarray profiles, and then a three‐snoRNA prognostic risk model consisting of SNORD1A, SNORA60, and SNORA66, was constructed using the LASSO method. We found that DLBCL patients could be stratified by the prognostic signature, and patients in the high‐risk cohorts tended to have a disappointing clinical outcome, as did ABC type DLBCL patients. This is consistent with the latest reports.^2^, ^3^ In addition, the risk signature could play an independent role, as a novel biomarker in the prognosis of DLBCL. A nomogram containing the risk signature, as well as other independent prognostic factors (age, stage and ECOG performance status) was constructed to visualize the risk model in clinical practice.

To date, predictive risk models based on mRNAs,^26^, ^41^ miRNAs,^42^, ^43^ lncRNAs,^44^, ^45^, ^46^ and circRNAs^47^ have been studied in DLBCL. However, no report exists on survival‐related snoRNA signatures in DLBCL patients. Our study was the first to systematically explore the clinical value of survival‐related snoRNAs in DLBCL, and provided evidence that SNORD1A, SNORA60, and SNORA66 could serve as prognostic risk models. In our selected snoRNAs, SNORD1A was a C/D box snoRNA, whereas SNORA60 and SNORA66 were H/ACA box snoRNAs. All three snoRNAs were detected in DLBCL tissues, suggesting the possibility of clinical detection in DLBCL patients. Additionally, we found potentially harmful roles of SNORD1A and SNORA60 in DLBCL with higher expression levels in high‐risk cohorts; moreover, up‐regulation of SNORD1A and SNORA60 correlated with an unfavorable outcome in DLBCL patients. However, SNORA66 was upregulated in the low‐risk group and served as a protective factor. Current studies have reported that SNORD1A is a survival‐related snoRNA in various carcinomas by analyzing snoRNA cancer genomic data from TCGA database.^48^ There is limited literature on the clinical value of SNORA60 and SNORA66 in tumors. Further investigations are needed to evaluate the roles of the three candidate snoRNAs in tumor carcinogenesis and the prognosis of DLBCL.

The AUC for this snoRNA model was 0.663 at 3 years, showing a certain predictive capacity after comparison with the published prognostic models, suggesting the possibility of clinical application in DLBCL patients. For instance, using the GSE11318 dataset, Xie et al. established a prognostic risk model using six related genes of RNA‐binding proteins, and achieved an the AUC value was 0.65 at 3 years.^49^ Zhang et al. constructed a risk model with an AUC of 0.564 based on alternative splicing events in DLBCL.^50^ Zhou et al. reported that the AUC of the immunoscore prognostic signature in DLBCL was 0.562.^51^ For a gene signature in DLBCL patients, Hu et al. obtained an AUC of 0.67 in their pharmacogenomic gene risk model.^25^ For some published snoRNA risk models, Cao et al. and Liu et al. have reported snoRNA signatures with an AUC of 0.664 at 3 years in bladder cancer, and an AUC of 0.657 at 3 years in sarcoma.^36^, ^39^ Summarily, as technology has been developing, this snoRNA model seems prospective and beneficial for improving the prognosis of DLBCL patients.

Currently, snoRNA host genes are known to play an oncogenic role in DLBCL; furthermore certain snoRNAs could advance the immune escape and progression of DLBCL by interacting with miRNAs.^16^, ^17^, ^18^, ^19^ However, understanding the mechanism of snoRNAs in DLBCL is rudimentary, thus limiting their application in clinical practice. Mechanism exploration in this research revealed that SNORD1A co‐expressed genes were mainly involved in pathways of pyrimidine metabolism, ribosome biogenesis in eukaryotes, RNA polymerase, purine metabolism, metabolic pathways, and oxidative phosphorylation, which belong to a series of processes related to ribosomes and mitochondria. Increasing evidence has demonstrated that snoRNAs can promote the development of cancer through the regulation of ribosome biogenesis, which plays essential roles in tumor cell growth.^52^, ^53^ Krogh et al. revealed that dysregulation of ribosomal modifications could be linked to DLBCL pathogenesis, including cell growth and tumor‐specific changes in DLBCL.^54^ Mitochondria dysfunction has also been verified as another vital factor in tumor proliferation and metastasis, and targeted drugs for certain mitochondria processes are regarded as promising strategies for patients with metastatic tumors.^55^, ^56^, ^57^ Summarily, we assumed that the selected SNORD1A co‐expressed genes might play crucial roles in DLBCL progression by influencing ribosomal and mitochondrial processes.

We obtained the potential transcription factors and hub genes for SNORD1A co‐expressed genes, and constructed a potential interactive network. Using ChIP‐seq data from the Cistrome Data Browser, we identified the binding sites in *p53* and several ribosomal protein‐correlated genes (*RPL10A*, *RPL12*, *RPL23*, and *RPS16*) in Mus musculus lymphoma tissue, and *ZIC2*‐*RPL12* in peritoneal effusion for human B cell lymphoma, suggesting their potential interaction in DLBCL. Tumor protein p53 (*TP53*) gene encodes a tumor suppressor protein that functions as a master regulator of diverse cellular activities, such as activation of transcription, apoptosis, and metabolism.^58^ Inhibition of apoptosis related to *p53* is recognized as a crucial factor in resistance to therapy for DLBCL.^59^ Ribosomal protein L10A (*RPL10A*), ribosomal protein L12 (*RPL12*), ribosomal protein L23 (*RPL23*) and ribosomal protein S16 (*RPS16*), encode ribosomal proteins (RPs) that are mostly implicated in protein synthesis.

RPs play extremely important roles in modulating *p53* function in cell cycle arrest and ribosome biogenesis in lymphoma, which is related to tumorigenesis and tumor development.^60^, ^61^, ^62^ *RPL23* has been reported to participate in a typical signaling pathway in malignancies, wherein *RPL23* blocks the inhibitory function of the oncoprotein murine double minute 2 (*MDM2*) to target and ubiquitinate p53 via the RP‐*MDM2*‐*p53* axis, further controlling tumor cell growth.^63^, ^64^, ^65^, ^66^ A study by Meng et al. verified that in dependence on MAPK/ERK kinase (MEK)/phosphoinositide 3‐kinase (PI3K) and mechanistic target of rapamycin (mTOR) pathways, RAS could increase *RPL23* expression and further cause cell cycle arrest regulated by *p53*.^67^ Watanabe et al. found that Glutamate Rich WD Repeat Containing 1 (*GRWD1*) could induce the proteolysis of *RPL23*, further decreasing *p53* expression, which is related to tumorigenesis.^68^ Current studies have illustrated that *RPL23* could modulate *p53*‐correlated cell apoptosis and cell cycle arrest through *MDM2*‐*p53* feedback loop modulation in in vitro experiments in carcinomas of the stomach,^69^, ^70^ colon,^71^ and lung,^72^ suggesting the involvement of the *RPL23*‐*MDM2*‐*p53* pathway checkpoint in malignancies. Here, we observed a possible interaction of *p53* and *RPL23* (Coordinate: chr11:97777525–97,782,438, Score: 1.4505) as well as of *p53* and *MDM2* (Coordinate: chr10:117688891–117,710,713, Score: 1.5943) in the mus musculus lymphoma tissue. As for correlation of *p53* and *RPL10A*, Jia et al. found that RPL10A is one of the critical components of eEF1B network in gastric cancer, potentially associated with the *p53* signaling pathway.^73^ Palasin et al. identified elevated expression levels of *TP53* in zebrafish with *RPL10A* deficiency.^74^ However, correlations of *p53*‐*RPL12*, *p53*‐*RPS16*, and *ZIC2*‐*RPL12* were found for the first time in this study. Thus, we speculated that the interaction of *p53* and *RPL23*, as well as *p53* and *RPL10A* is of therapeutic value in DLBCL, but further experimental evidence is required to verify this.

In this study, *MYC* and *RPL10A* were recognized as the most mutated genes for SNORD1A co‐expression in DLBCL. *MYC* acts as a transcription factor associated with cell cycle, biosynthesis, and apoptosis. Close links between *MYC* mutations and hematopoietic tumors are well‐known. Double‐hit DLBCL contains *MYC* translocations and translocations of *BCL2* or *BCL6*, with an extremely disappointing outcome, making *MYC* an attractive therapeutic target.^75^ Currently, treatment strategies targeting the biosynthesis of *MYC* that affect tumor cells of DLBCL have been reported.^75^ Drugs based on Rocaglates have been gradually applied in treatment of lymphomas with *MYC* mutations, with promising therapeutic value.^76^ *RPL10A* belongs to the L1P family of ribosomal proteins. Derenzini et al. found that patients with RP mutations tend to have statistically worse clinical outcomes than patients with wild‐type TP53,^77^ of which *RPL10A* mutations were identified. A correlation between *p53* and *RPL10A* was also observed in our study. Thus, we presumed that SNORD1A co‐expressed genes might produce effects in DLBCL through *MYC* and *RPL10A* mutations, of which *RPL10A* mutations might interact with *p53*, providing novel insights into the molecular mechanism of DLBCL.

Generally, a novel three‐snoRNA signature was firstly mentioned in this study, providing new ideas for the clinical treatment and management of DLBCL patients. However, this research has some limitations that the design is retrospective, and the sensitivity and specificity of the risk model are not sufficiently high. Moreover, no experiments were conducted to verify the mechanism of SNORD1A co‐expressed genes. Therefore, multicenter large cohort data and further experiments are necessary to validate our findings and increase the possibilities of seeking more effective snoRNA biomarkers for prognosis of DLBCL patients.

## 5 CONCLUSION

A comprehensive analysis was performed to select significant survival‐related snoRNAs in DLBCL. Collectively, a prognostic signature based on three snoRNAs (SNORD1A, SNORA60, and SNORA66) was established for DLBCL patients. The three‐snoRNA signature and certain clinicopathological parameters (age, stage and ECOG performance status) were considered as independent risk factors. Also, we found snoRNA co‐expressed genes that were mostly involved in ribosome‐correlated pathways and processes, and the interaction of ribosomal proteins (*RPL23*, *RPL10A*) and p53. The current study is the first to determine the prognostic significance of snoRNAs in DLBCL, develop a novel scoring system for clinical application, and explore the potential molecular mechanisms underlying DLBCL. Nonetheless, more clinical data and experiments are needed for further validation of this risk model.

## AUTHOR CONTRIBUTIONS

Li MW contributed to the study conception, data analysis and manuscript writing. Huang FX participated in the study supervision and manuscript writing. Li MW and Huang FX should be considered as joint first author. Xie ZC involved in data analysis. Hong HY and Xu QY collected and checked the data. Peng ZG was responsible for conception and design of the study.

## FUNDING INFORMATION

This research was supported by the Appropriate Hygiene Technology Development, Popularization and Application Project in Guangxi, China [S2020037], as well as projects funded by the China Anti‐cancer Association (CORP‐117), and the International Scientific Exchange Foundation of China [Z2020LGX001].

## CONFLICT OF INTEREST

No conflict of interest was declared by the authors.

## ETHICAL APPROVAL STATEMENT

Not applicable.

## CLINICAL TRIAL REGISTRATION NUMBER

Not applicable.

## Supporting information


Figure S1–S2

Table S1–S2

## Data Availability

The datasets supporting the current study are available in GEO database (https://www.ncbi.nlm.nih.gov/geo).
