# Supplementary figures and images for: Identification of three small nucleolar RNAs (snoRNAs) as potential prognostic markers in diffuse large B‐cell lymphoma
Source: Cancer Med. 2022 Aug 16;12(3):3812–29. doi: 10.1002/cam4.5115 (PMC9939161; doi:10.1002/cam4.5115)

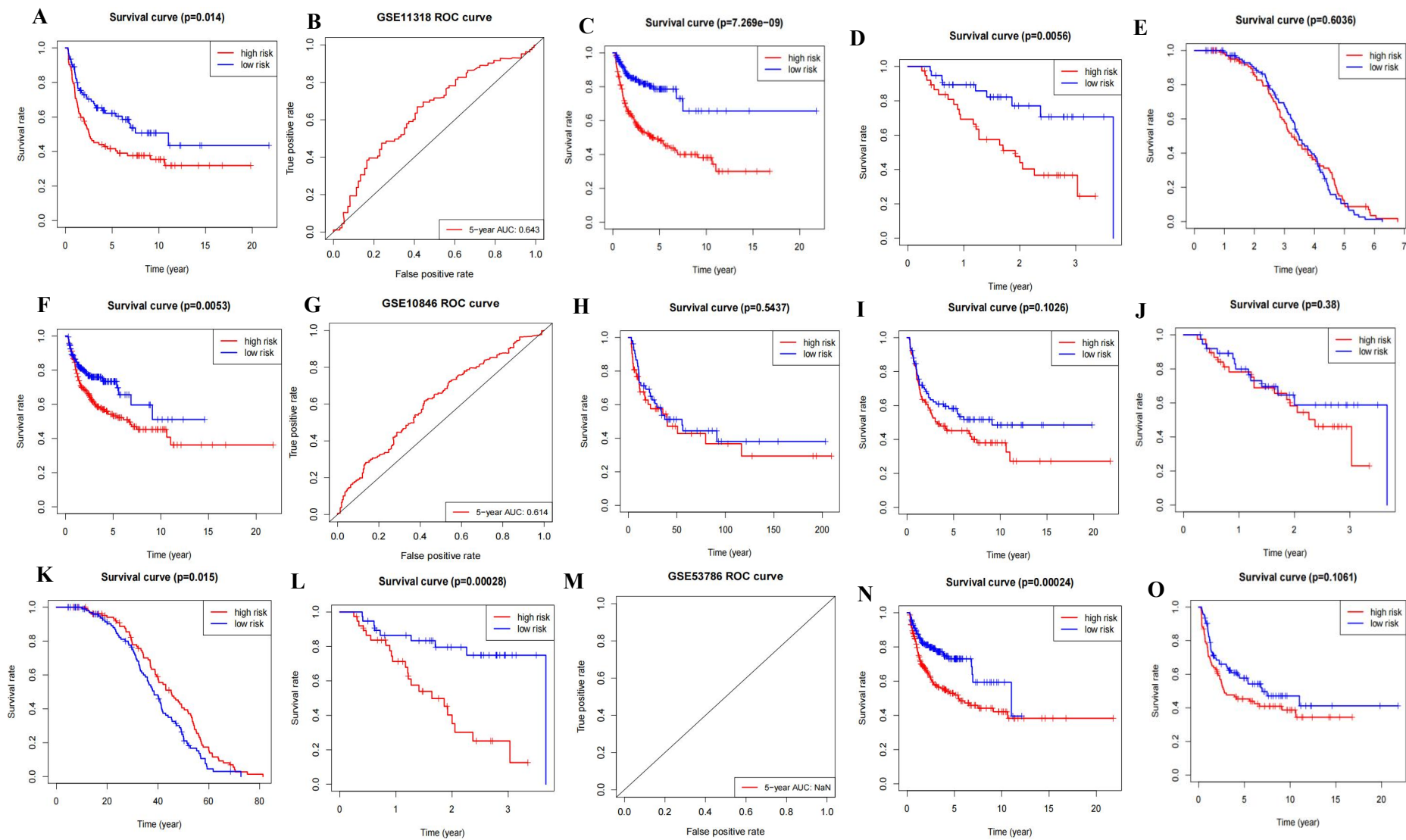

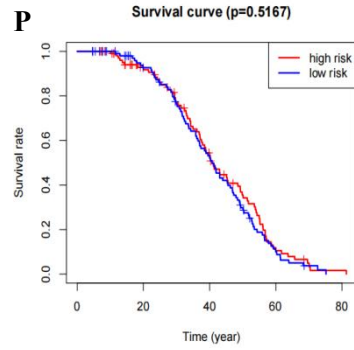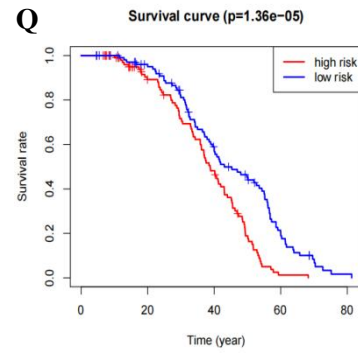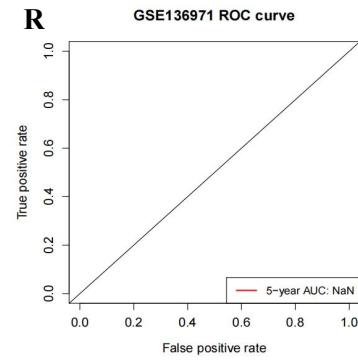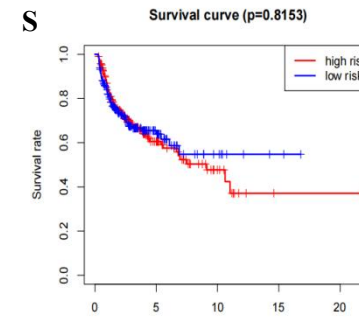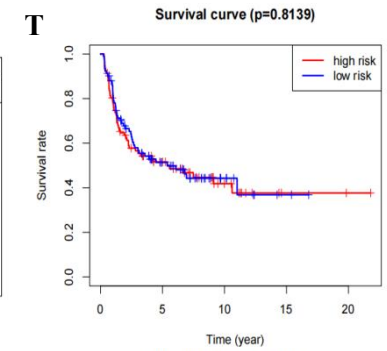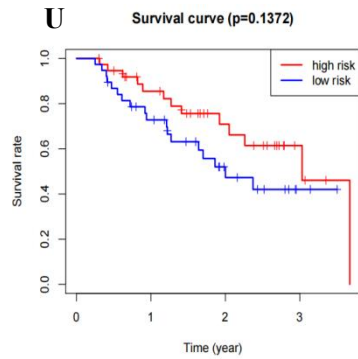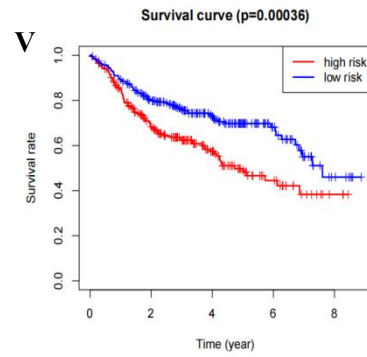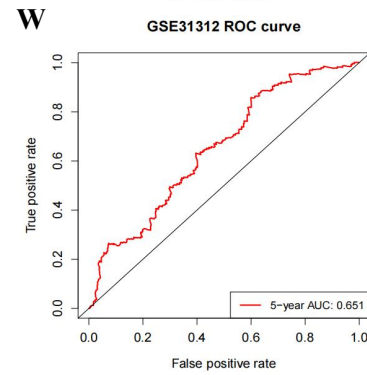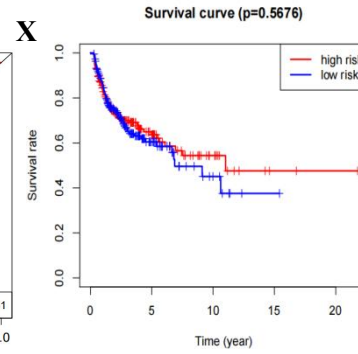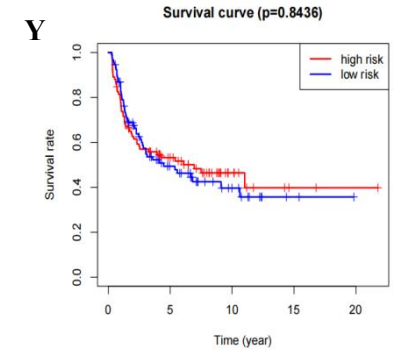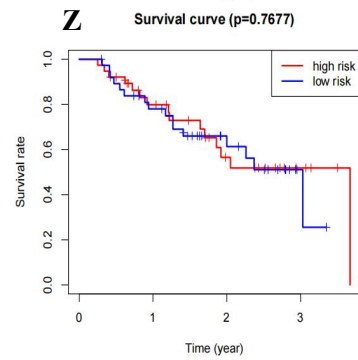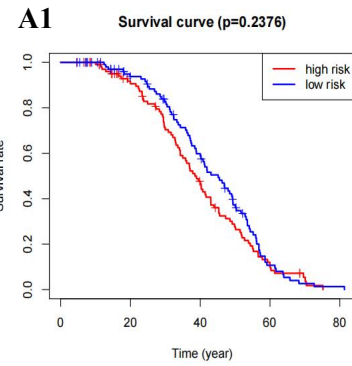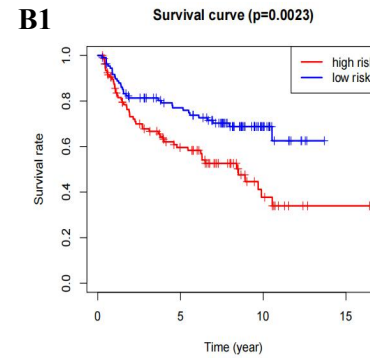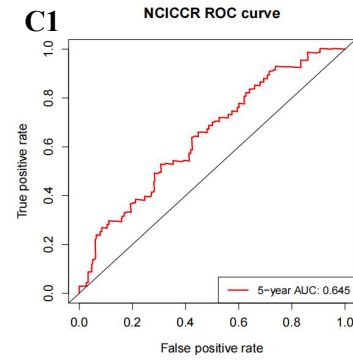

**A**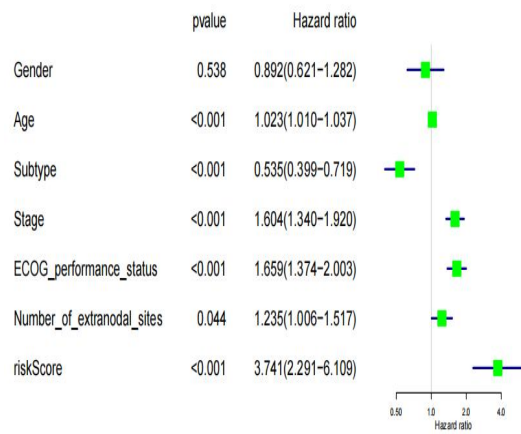**B**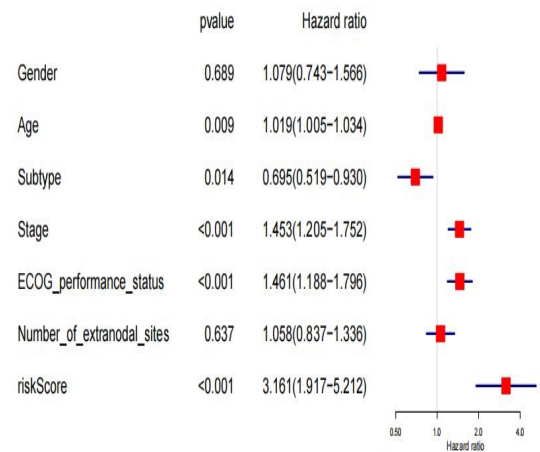**C**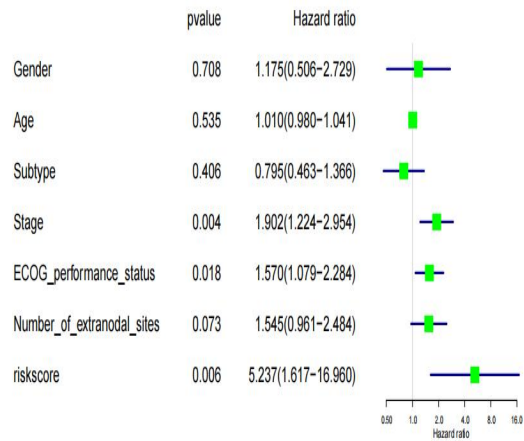**D**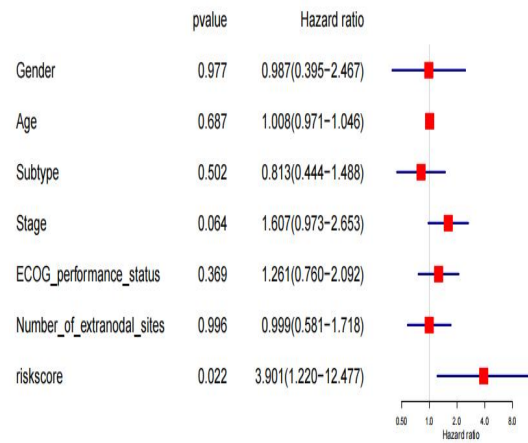

Supplement: Supplementary file 1 — Figure S1–S2 [file CAM4-12-3812-s002.pdf]
