# Supplementary material for: Identification of three small nucleolar RNAs (snoRNAs) as potential prognostic markers in diffuse large B‐cell lymphoma
Source: Cancer Med. 2022 Aug 16;12(3):3812–29. doi: 10.1002/cam4.5115 (PMC9939161; doi:10.1002/cam4.5115)
Supplement: Supplementary file 2 — Table S1–S2 [file CAM4-12-3812-s001.docx]

**Supplementary Table 1. A total of 751 human** **snoRNAs were acquired from** **the SnoDB online database.**

| AC006023.2 | SCARNA17 | SNODB2066 | snoMe28S-Am2634 | SNORA118 | SNORA1B | SNORA30 | SNORA41B | SNORA57 |
| --- | --- | --- | --- | --- | --- | --- | --- | --- |
| AC011452.6 | SCARNA18 | SNODB2067 | snoR1 | SNORA119 | SNORA2 | SNORA30B | SNORA42 | SNORA58 |
| AC018563.7 | SCARNA18B | SNODB2068 | SNORA1 | SNORA11B | SNORA20 | SNORA31 | SNORA43 | SNORA58B |
| AC087632.10 | SCARNA2 | SNODB2069 | SNORA10 | SNORA11C | SNORA20B | SNORA31B | SNORA43L1 | SNORA59A |
| AC092015.2 | SCARNA20 | SNODB2070 | SNORA100 | SNORA11D | SNORA20L4 | SNORA32 | SNORA43L3 | SNORA59B |
| AC096917.3 | SCARNA21 | SNODB2071 | SNORA101A | SNORA11E | SNORA21 | SNORA33 | SNORA44 | SNORA5A |
| AC138972.8 | SCARNA21B | SNODB2072 | SNORA101B | SNORA11F | SNORA21B | SNORA33L1 | SNORA46 | SNORA5B |
| ACA59 | SCARNA22 | SNODB2073 | SNORA103 | SNORA11G | SNORA22 | SNORA35 | SNORA47 | SNORA5C |
| ACA64 | SCARNA23 | SNODB2074 | SNORA104 | SNORA12 | SNORA22B | SNORA35B | SNORA48 | SNORA6 |
| ACEA_U3 | SCARNA24 | SNODB2075 | SNORA105A | SNORA120 | SNORA22C | SNORA36 | SNORA48B | SNORA60 |
| AL023281.1 | SCARNA26A | SNODB2076 | SNORA105B | SNORA13 | SNORA23 | SNORA36A | SNORA48L10 | SNORA61 |
| AL137247.14 | SCARNA26B | SNODB2077 | SNORA105C | SNORA14 | SNORA24 | SNORA36B | SNORA48L3 | SNORA62 |
| AL138745.19 | SCARNA27 | SNODB2078 | SNORA107 | SNORA14A | SNORA24B | SNORA36C | SNORA49 | SNORA63 |
| AL589947.3 | SCARNA28 | SNODB2079 | SNORA108 | SNORA14B | SNORA25 | SNORA37 | SNORA5 | SNORA63B |
| AP000818.4 | SCARNA3 | SNODB2080 | SNORA109 | SNORA15 | SNORA25B | SNORA38 | SNORA50 | SNORA63C |
| RNU105B | SCARNA4 | SNODB2081 | SNORA10B | SNORA15B-1 | SNORA25L6 | SNORA38B | SNORA50A | SNORA63D |
| RNU105C | SCARNA5 | SNODB2082 | SNORA11 | SNORA15B-2 | SNORA25L8 | SNORA3A | SNORA50B | SNORA63E |
| SCARNA1 | SCARNA6 | SNODB2083 | SNORA110 | SNORA16 | SNORA26 | SNORA3B | SNORA50C | SNORA63L3 |
| SCARNA10 | SCARNA7 | SNODB2084 | SNORA111 | SNORA16A | SNORA27 | SNORA3C | SNORA50D | SNORA64 |
| SCARNA11 | SCARNA8 | SNODB2085 | SNORA112 | SNORA16B | SNORA28 | SNORA4 | SNORA51 | SNORA65 |
| SCARNA12 | SCARNA9 | SNODB2086 | SNORA113 | SNORA17 | SNORA29 | SNORA40 | SNORA52 | SNORA66 |
| SCARNA13 | SCARNA9L | SNODB2087 | SNORA114 | SNORA17A | SNORA2A | SNORA40B | SNORA53 | SNORA67 |
| SCARNA14 | SCARNA9L1 | SNODB2088 | SNORA115 | SNORA17B | SNORA2B | SNORA40C | SNORA54 | SNORA67L1 |
| SCARNA15 | sno_ZL2 | SNODB2089 | SNORA116 | SNORA18 | SNORA2C | SNORA40L2 | SNORA55 | SNORA67L3 |
| SCARNA16 | SNODB2065 | snoMBII-202 | SNORA117 | SNORA19 | SNORA3 | SNORA41 | SNORA56 | SNORA68 |
| SNORA71D | SNORA7B | SNORD101 | SNORD113-7 | SNORD114-29 | SNORD115-22 | SNORD115-45 | SNORD116-23 | SNORD124 |
| SNORA71E | SNORA8 | SNORD102 | SNORD113-8 | SNORD114-3 | SNORD115-23 | SNORD115-46 | SNORD116-24 | SNORD125 |
| SNORA72 | SNORA80A | SNORD103 | SNORD113-9 | SNORD114-30 | SNORD115-24 | SNORD115-47 | SNORD116-25 | SNORD126 |
| SNORA72L5 | SNORA80B | SNORD103A | SNORD114-1 | SNORD114-31 | SNORD115-25 | SNORD115-48 | SNORD116-26 | SNORD127 |
| SNORA73 | SNORA80C | SNORD103B | SNORD114-10 | SNORD114-4 | SNORD115-26 | SNORD115-5 | SNORD116-27 | SNORD128 |
| SNORA73A | SNORA80D | SNORD103C | SNORD114-11 | SNORD114-5 | SNORD115-27 | SNORD115-6 | SNORD116-28 | SNORD129 |
| SNORA73B | SNORA80E | SNORD104 | SNORD114-12 | SNORD114-6 | SNORD115-28 | SNORD115-7 | SNORD116-29 | SNORD12B |
| SNORA74 | SNORA81 | SNORD105 | SNORD114-13 | SNORD114-7 | SNORD115-29 | SNORD115-8 | SNORD116-3 | SNORD12C |
| SNORA74A | SNORA84 | SNORD105B | SNORD114-14 | SNORD114-8 | SNORD115-3 | SNORD115-9 | SNORD116-30 | SNORD13 |
| SNORA74B | SNORA86 | SNORD107 | SNORD114-15 | SNORD114-9 | SNORD115-30 | SNORD116 | SNORD116-4 | SNORD130 |
| SNORA74C-1 | SNORA87 | SNORD108 | SNORD114-16 | SNORD115 | SNORD115-31 | SNORD116-1 | SNORD116-5 | SNORD131 |
| SNORA74C-2 | SNORA88 | SNORD109A | SNORD114-17 | SNORD115-1 | SNORD115-32 | SNORD116-10 | SNORD116-6 | SNORD132 |
| SNORA74D | SNORA89 | SNORD109B | SNORD114-18 | SNORD115-10 | SNORD115-33 | SNORD116-11 | SNORD116-7 | SNORD133 |
| SNORA75 | SNORA9 | SNORD11 | SNORD114-19 | SNORD115-11 | SNORD115-34 | SNORD116-12 | SNORD116-8 | SNORD134 |
| SNORA75B | SNORA90 | SNORD110 | SNORD114-1L1 | SNORD115-12 | SNORD115-35 | SNORD116-13 | SNORD116-9 | SNORD135 |
| SNORA75L3 | SNORA91 | SNORD111 | SNORD114-2 | SNORD115-13 | SNORD115-36 | SNORD116-14 | SNORD116-9L1 | SNORD136 |
| SNORA75L4 | SNORA92 | SNORD111B | SNORD114-20 | SNORD115-14 | SNORD115-37 | SNORD116-15 | SNORD117 | SNORD137 |
| SNORA75L6 | SNORA93 | SNORD112 | SNORD114-21 | SNORD115-15 | SNORD115-38 | SNORD116-16 | SNORD118 | SNORD138 |
| SNORA76 | SNORA94 | SNORD113 | SNORD114-22 | SNORD115-16 | SNORD115-39 | SNORD116-17 | SNORD118L4 | SNORD139 |
| SNORA77 | SNORA95 | SNORD113-1 | SNORD114-23 | SNORD115-17 | SNORD115-4 | SNORD116-18 | SNORD119 | SNORD13B-1 |
| SNORA77B | SNORA98 | SNORD113-2 | SNORD114-24 | SNORD115-18 | SNORD115-40 | SNORD116-19 | SNORD11B | SNORD13B-2 |
| SNORA78 | SNORA99 | SNORD113-3 | SNORD114-25 | SNORD115-19 | SNORD115-41 | SNORD116-2 | SNORD12 | SNORD13C |
| SNORA79 | SNORA9B | SNORD113-4 | SNORD114-26 | SNORD115-2 | SNORD115-42 | SNORD116-20 | SNORD121A | SNORD13D |
| SNORA79B | SNORD10 | SNORD113-5 | SNORD114-27 | SNORD115-20 | SNORD115-43 | SNORD116-21 | SNORD121B | SNORD13E |
| SNORA7A | SNORD100 | SNORD113-6 | SNORD114-28 | SNORD115-21 | SNORD115-44 | SNORD116-22 | SNORD123 | SNORD13F |
| SNORD152 | SNORD175 | SNORD3-L11 | SNORD3-L38 | SNORD36A | SNORD3L4 | SNORD50A | SNORD63 | SNORD77 |
| SNORD153 | SNORD18 | SNORD3-L12 | SNORD3-L39 | SNORD36B | SNORD3L6 | SNORD50B | SNORD63B | SNORD77B |
| SNORD154 | SNORD18A | SNORD3-L13 | SNORD3-L4 | SNORD36C | SNORD3L7 | SNORD51 | SNORD64 | SNORD78 |
| SNORD155 | SNORD18B | SNORD3-L14 | SNORD3-L40 | SNORD37 | SNORD3P3 | SNORD52 | SNORD65 | SNORD79 |
| SNORD156 | SNORD18C | SNORD3-L15 | SNORD3-L41 | SNORD38 | SNORD41 | SNORD53 | SNORD65B | SNORD8 |
| SNORD157 | SNORD19 | SNORD3-L16 | SNORD3-L42 | SNORD38A | SNORD42 | SNORD53B | SNORD65C | SNORD80 |
| SNORD158 | SNORD19B | SNORD3-L18 | SNORD3-L43 | SNORD38B | SNORD42A | SNORD54 | SNORD66 | SNORD80L1 |
| SNORD159 | SNORD19C | SNORD3-L19 | SNORD3-L44 | SNORD38C | SNORD42B | SNORD55 | SNORD66L1 | SNORD81 |
| SNORD15A | SNORD1A | SNORD3-L20 | SNORD3-L46 | SNORD38D | SNORD43 | SNORD55L1 | SNORD67 | SNORD81L4 |
| SNORD15B | SNORD1B | SNORD3-L21 | SNORD3-L47 | SNORD39 | SNORD43L1 | SNORD56 | SNORD68 | SNORD82 |
| SNORD16 | SNORD1C | SNORD3-L22 | SNORD3-L48 | SNORD3A | SNORD44 | SNORD56B | SNORD69 | SNORD83 |
| SNORD160 | SNORD2 | SNORD3-L23 | SNORD3-L5 | SNORD3B-1 | SNORD45 | SNORD57 | SNORD7 | SNORD83A |
| SNORD161 | SNORD20 | SNORD3-L24 | SNORD3-L7 | SNORD3B-2 | SNORD45A | SNORD58 | SNORD70 | SNORD83B |
| SNORD162 | SNORD21 | SNORD3-L27 | SNORD3-L8 | SNORD3C | SNORD45AL1 | SNORD58A | SNORD70B | SNORD84 |
| SNORD163 | SNORD22 | SNORD3-L28 | SNORD3-L9 | SNORD3D | SNORD45B | SNORD58B | SNORD71 | SNORD86 |
| SNORD164 | SNORD23 | SNORD3-L29 | SNORD30 | SNORD3E | SNORD45C | SNORD58C | SNORD72 | SNORD87 |
| SNORD165 | SNORD24 | SNORD3-L3 | SNORD31 | SNORD3F | SNORD46 | SNORD59 | SNORD73A | SNORD88 |
| SNORD166 | SNORD25 | SNORD3-L30 | SNORD31B | SNORD3G | SNORD47 | SNORD59A | SNORD73B | SNORD88A |
| SNORD167 | SNORD26 | SNORD3-L31 | SNORD32A | SNORD3H | SNORD48 | SNORD59B | SNORD74 | SNORD88B |
| SNORD168 | SNORD27 | SNORD3-L32 | SNORD32B | SNORD3I | SNORD49A | SNORD6 | SNORD74B | SNORD88C |
| SNORD169 | SNORD28 | SNORD3-L33 | SNORD33 | SNORD3J | SNORD49B | SNORD60 | SNORD74L1 | SNORD88CL1 |
| SNORD17 | SNORD28B | SNORD3-L34 | SNORD34 | SNORD3K | SNORD4A | SNORD61 | SNORD74L2 | SNORD89 |
| SNORD170 | SNORD29 | SNORD3-L35 | SNORD35A | SNORD3L1 | SNORD4B | SNORD62 | SNORD74L6 | SNORD9 |
| SNORD172 | SNORD3 | SNORD3-L36 | SNORD35B | SNORD3L2 | SNORD5 | SNORD62A | SNORD75 | SNORD90 |
| SNORD173 | SNORD3-L10 | SNORD3-L37 | SNORD36 | SNORD3L3 | SNORD50 | SNORD62B | SNORD76 | SNORD91A |
| Z73421.1 | SNORA68B | SNORA68L2 | SNORA69 | SNORA7 | SNORA70 | SNORA70B | SNORA70BL3 | SNORA70BL5 |
| SNORA70C | SNORA70D | SNORA70E | SNORA70EL1 | SNORA70EL4 | SNORA70EL5 | SNORA70F | SNORA70FL2 | SNORA70G |
| SNORA70H | SNORA70I | SNORA70J | SNORA70L2 | SNORA71 | SNORA71A | SNORA71B | SNORA71C | SNORD13G |
| SNORD13H | SNORD13I | SNORD13J | SNORD13P1 | SNORD13P3 | SNORD14 | SNORD140 | SNORD141A | SNORD141B |
| SNORD142 | SNORD143 | SNORD144 | SNORD145 | SNORD146 | SNORD147 | SNORD148 | SNORD149 | SNORD14A |
| SNORD14B | SNORD14C | SNORD14D | SNORD14E | SNORD150 | SNORD151 | SNORD91B | SNORD92 | SNORD93 |
| SNORD94 | SNORD95 | SNORD96A | SNORD96B | SNORD97 | SNORD98 | SNORD99 | snosnR60_Z15 | snosnR66 |
| snoU109 | snoU13 | snoU18 | snoU2_19 | snoU2-30 | snoU83B | snoZ196 | snoZ278 | snoZ40 |
| snoZ6 | TERC | U3 | U8 |  |  |  |  |  |

**Supplementary Table 2. Establishment of a snoRNA-based risk signature for DLBCL.**

| Training datasets | Platform | Cases | snoRNAs in risk model | P value of  K-M analysis | AUC | Risk formula | Validating  datasets |
| --- | --- | --- | --- | --- | --- | --- | --- |
| GSE11318 | GPL570 | 184 | SNORD1A, SNORA60  SNORA66 | p=0.0140 | 0.643 | (SNORD1A)*(0.3709)+(SNORA60)*  (0.4059)+(SNORA66)*(-0.1106) | GSE10846 GSE53786  GSE136971 |
| GSE10846 | GPL570 | 380 | SNORA66, SNORD35B  SNORA70 | p=0.0053 | 0.614 | (SNORA66)*(-0.1162)+(SNORD35B)*(-0.3024)+(SNORA70)*(0.3196) | NA |
| GSE53786 | GPL570 | 108 | SNORD84, SNORD4A SNORD1A | p＜0.001 | NaN | (SNORD84)*(0.3569)+(SNORD4A)*  (0.2717)+(SNORD1A)*(0.6287) | NA |
| GSE136971 | GPL570 | 214 | SNORA67, SNORA11D  SNORA37 | p＜0.001 | NaN | (SNORA67)*(0.1545)+(SNORA11D)*  (0.1609)+(SNORA37)*(-0.2634) | NA |
| GSE31312 | GPL570 | 470 | SNORA68, SNORD8  SCARNA13 | p＜0.001 | 0.651 | (SNORA68)*(-1.3187)+(SNORD8)*  (-1.4198)+(SCARNA13)*(1.8557) | NA |
| NCICCR | - | 218 | SNORD104, SNORD51  SNORD116-18 | p=0.0023 | 0.645 | (SNORD104)*(0.0122)+(SNORD51)*  (-0.1513)+(SNORD116-18)*(0.5224) | NA |

Note: NCICCR: the National Cancer Institute Center for Cancer Research; p <0.05 was regarded to be statistically significant; NaN: AUC value can not be calculated; NA: The snoRNAs of the model could not be found or validated in other datasets.
